# Clinical characteristics and antimicrobial susceptibility of *Bacillus cereus* blood stream infections

**DOI:** 10.1186/s12941-015-0104-2

**Published:** 2015-09-15

**Authors:** Mahoko Ikeda, Yuka Yagihara, Keita Tatsuno, Mitsuhiro Okazaki, Shu Okugawa, Kyoji Moriya

**Affiliations:** Department of Infection Control and Prevention, Faculty of Medicine, The University of Tokyo, 7-3-1 Hongo, Bunkyo, Tokyo 113-8655 Japan; Department of Medical Technology, School of Health Sciences, Tokyo University of Technology, 5-23-22 Nishikamata, Ota, Tokyo 144-8535 Japan

**Keywords:** *Bacillus cereus*, Blood stream infection, Empirical therapy, Susceptibility

## Abstract

**Background:**

*Bacillus cereus* is one of the pathogens causing nosocomial bloodstream infections (BSIs). However, few reports have documented the antimicrobial susceptibility and clinical characteristics of *Bacillus cereus* BSI and the importance of empirical therapy. The aim of this study was to investigate the clinical characteristics and antimicrobial susceptibility of *B. cereus* isolates from patients with BSI and to analyze the impact of appropriate empirical therapy on the outcome of patients with *B. cereus* BSI.

**Methods:**

All adult cases of bacteremia between April 2003 and March 2012 in a teaching hospital in Tokyo, Japan were reviewed retrospectively. Clinical data were collected from the patients’ medical records and charts. Antimicrobial susceptibility testing was performed by broth microdilution method. The patients with *B. cereus* BSI were divided into an appropriate empirical therapy group and an inappropriate empirical therapy group. The primary outcome was all-cause mortality at 4 weeks after the start of BSI. The secondary outcome was early defervescence within 2 days after starting empirical therapy.

**Results:**

There were 29 *B. cereus* bloodstream infection cases. No vancomycin, gentamicin, and imipenem-resistant isolates were found. However, 65.5 % were resistant to clindamycin and 10.3 % were resistant to levofloxacin. The main etiology was venous catheter-related (69 %). All-cause mortality at 4 weeks was not significantly different between the appropriate empirical therapy group (9 cases) and the inappropriate group (20 cases) in this study. However, early defervescence within 2 days after starting empirical therapy was significantly different (*p* = 0.032).

**Conclusions:**

The BSI of *B.cereus* is mostly caused by venous catheter-related infections. Appropriate empirical therapy is important to achieve early clinical resolution in *B. cereus* BSI. Vancomycin is one of the appropriate selections of empirical therapy for *B. cereus* BSI.

## Background

The *Bacillus cereus* group contains six species, *B. cereus*, *B. thuringiensis*, *B. weihenstephanensis*, *B. mycoides*, *B. pseudomycoides*, and *B. anthracis* [1]. The most prevalent human pathogen in the *B. cereus* group is *B. cereus* [1]. The automated identification system in diagnostic laboratories cannot distinguish between *B. cereus* and *B. thuringiensis,* because they are genetically similar species. However, *B. thuringiensis* is an extremely rare human pathogen and is basically an insect pathogen [2].

*B. cereus* is a spore-forming Gram-positive bacillus that exists ubiquitously in soil, marine environments, vegetables, the intestinal tracts of invertebrates, and human skin [3]. The organism is a common pathogen in food poisoning [3]. Invasive *B. cereus* infections such as bacteremia, pneumonia [4], eye infection [5], central nervous system (CNS) infections [6, 7], and soft tissue infections [8, 9] have been reported in hospital settings. More information about the clinical characteristics of *B. cereus* bloodstream infection (BSI) is urgently needed to improve the treatment of patients with *B. cereus* infection.

BSIs are important causes of morbidity and mortality. Appropriate empirical therapy should be started immediately after BSI is identified [10]. Recent reports have analyzed risk factors for *B. cereus* BSI, such as prior antimicrobial treatment [11, 12], reuse of contaminated towels [13, 14], central venous catheter insertions [12, 15], and hematological malignancy [16]. Fatal outcomes have been related to neutropenia [6], delays in treatment [17], and CNS symptoms [15, 17].

Inappropriate antibiotic therapy has been shown to be predictive of higher mortality rates in patients with bacteremia compared to appropriate therapy [10]. The antimicrobial agents for the empirical therapy should be selected according to the antimicrobial susceptibility of the pathogen. However, few studies have reported on the clinical characteristics of *B. cereus* BSI and the importance of empirical therapy [17, 18]. The aim of this study was therefore to investigate the antimicrobial susceptibility of clinical *B. cereus* isolates from patients with BSI and to analyze the impact of appropriate empirical therapy on clinical outcomes in patients with *B. cereus* BSI.

## Methods

### Study setting and patients

All cases of bacteremia that occurred between April 2003 and March 2012 in the microbial blood culture database of the University of Tokyo Hospital, a 1217-bed tertiary-care teaching hospital in Tokyo, Japan, were retrospectively reviewed. Patients with *B. cereus* bacteremia were divided into appropriate empirical therapy and inappropriate empirical therapy groups.

### Data collection and definitions

All adult (age ≥18 years) patients from whose blood cultures *B.cereus* was isolated were enrolled. Bacteremia caused by *B. cereus* was defined as: (1) two or more positive blood cultures collected in one day; or (2) positive blood cultures collected on more than two consecutive days. Untreated cases and cases that received oral antimicrobial therapy were excluded, as were patients who were afebrile despite positive blood cultures for *B. cereus*.

Patients’ data, clinical symptoms, laboratory results, antibiotics used, in-hospital mortality due to any cause, presence of blood cultures that became negative, and microbiological data were collected from the clinical charts and the laboratory database. Patients’ data included age, sex, underlying disease (diabetes mellitus, cirrhosis, malignancy, immunosuppressants, and neutropenia), and implanted medical devices such as central venous catheters, biliary stents, and artificial joints. Clinical symptoms included fever, suspected focus of infection, and complications with BSI. Laboratory data were white blood cell counts, neutrophil counts, C-reactive protein and albumin levels. Data of antibiotics used before and after blood culture samples were obtained were also reviewed.

Fever was defined as a body temperature ≥37.5 °C. When a body temperature of <37.5 °C was maintained for >24 h, the patient was defined as afebrile. Body temperature was measured at least 3 times a day (at 08:00, 14:00, and 20:00). A complication was considered a focus of infection accompanied by *B. cereus* BSI.

Empirical therapy was defined as the antibiotics received on the first day of therapy for the BSI. Appropriate antimicrobial therapy was defined as systemic administration of at least one antimicrobial agent to which the *B. cereus* isolate proved susceptible in vitro.

### Microbiological methods

Blood culture specimens were inoculated into BACTEC standard culture bottles in a BACTEC 9000 system (Becton, Dickinson and Company, Franklin Lakes, NJ, USA). All isolates were identified as *B. cereus*/*B. thuringiensis* using a VITEK2 system with the BCL card (SYSMEX bioMerieux, Tokyo, Japan). Antimicrobial susceptibility was determined with the Walkaway system and the standard criteria of the Clinical and Laboratory Standards Institute (CLSI) guide. The breakpoints against *B. cereus* in the CLSI guideline M45A2E [19] were used for the following agents: ampicillin, cefazolin, cefotaxime, caftazidime, imipenem, vancomycin, amikacin, gentamicin, erythromycin, levofloxacin, clindamycin, chloramphenicol and rifampin. For other antimicrobials, the breakpoints for *Staphylococcus* spp. in the CLSI guideline M 100-S22 [20] and S24 [21] were used [22].

### Clinical outcomes

The primary outcome was all-cause mortality at 4 weeks after onset of bacteremia. The secondary outcome was early defervescence within 2 days after starting empirical therapy.

### Statistical analysis

Fisher’s exact test was used for analysis of categorical data. Non-parametric data were analyzed using the Mann–Whitney U test. Values of *P* < 0.05 were considered significant. All statistical analyses were performed using JMP Pro version 10 software (SAS Institute, Cary, NC, USA).

## Results

### Patients’ characteristics

A total of 5894 cases with positive blood cultures was identified over the 9-year study period. Of these, *B.cereus* was isolated in 203 cases. One hundred seventy one cases were excluded, because they had one *B. cereus*-positive blood culture. Three cases with more than two *B. cereus*-positive blood cultures were also excluded, because one case was untreated, one case was treated with oral antibiotics, and one case was afebrile. After applying the exclusion criteria, 29 cases (14.3 % of cases in which *B. cereus* was detected, 0.49 % of all culture-positive cases) were included in this study. The patients’ clinical characteristics are shown in Table 1. The mean age was 65.3 years (range 18–89 years), and 55.2 % were male. The main etiology was venous catheter-related (69 %). The appropriate and inappropriate empirical therapy groups had similar baseline characteristics, sources of infection, and comorbidities that could significantly affect the clinical outcome. Complications except focuses of infection cited in Table 1 was endophthalmitis (1 case) occurred in the inappropriate empirical therapy group. In laboratory results, patients in the appropriate therapy group had a tendency to have higher white blood cell counts than patients in the inappropriate therapy group.

**Table 1:** Characteristics of patients with *Bacillus cereus* bloodstream infection

Variable	All patients (n = 29)	Empirical therapy that was deemed		*P* value
		Appropriate (n = 9)	Inappropriate (n = 20)	
Age, years (median, range)	65.3 (18–89)	68 (18–89)	65 (47–83)	0.743
Sex (male/female)	16/13	4/5	12/8	0.688
No. of patients with comorbidity				
Diabetes mellitus	8	3	5	0.675
Malignancy	15	4	11	0.700
Liver cirrhosis	4	1	3	1.00
Immunosuppressant	5	2	3	0.633
Neutropenia	4	0	4	0.280
No. of patients with implanted device				
Central venous catheter	6	1	5	0.633
Peripheral venous catheter	22	8	14	0.382
Others	7	3	4	0.642
Source of BSI				
Total catheter-related infection	20	7	13	0.675
Peripheral blood catheter	15	6	9	0.427
Central venous catheter	5	1	4	1.00
Others^a^	5	0	5	0.153
Unknown	4	2	2	0.568
Laboratory data (average, range)				
White blood cell count (/μL)	7497 (100–22,200)	9630 (1800–22,200)	6537 (100–16,100)	0.052
C-reactive protein (g/dL)	4.19 (0.04–16.6)	3.79 (0.04–16.6)	3.34 (0.17–13.7)	0.514
Albumin (g/dL)	3.29 (2.10–4.20)	2.71 (2.10–3.90)	3.45 (2.40–4.20)	0.206

^a^Other sources of BSI in the inappropriate empirical therapy group include febrile neutropenia (2 cases), possible infective endocarditis according to modified Duke’s criteria (2 cases), and peritonitis (1 case)

### Antimicrobial susceptibility

All isolates showed sensitivity to vancomycin, gentamicin, and imipenem. However, 48.3–100 % of isolates were resistant to cephalosporins, 65.5 % were resistant to clindamycin, and 10.3 % were resistant to levofloxacin (Table 2).

**Table 2:** Antimicrobial susceptibility of major antibiotics against *Bacillus cereus*

Antimicrobial agent	n	MIC^a^ (mg/dL)			Interpretation n (%)		
		Range	50 %^b^	90 %^b^	Susceptible	Interpretive	Resistant
Vancomycin	29	≤2	≤2	≤2	29 (100)	0	0
Imipenem	29	≤0.5 to 4	≤0.5	≤0.5	29 (100)	0	0
Gentamicin	29	≤2 to 4	≤2	4	29 (100)	0	0
Amikacin	26	≤16	≤16	≤16	26 (100)	0	0
Linezolid	25	≤0.25 to 2	1	1	25 (100)		
Chloramphenicol	22	2 to 4	4	4	22 (100)	0	0
Rifampin	15	≤1	≤1	≤1	15 (100)	0	0
Levofloxacin	29	≤0.12 to >16	≤0.5	≤0.5	26 (89.7)	1 (3.4)	2 (6.9)
Clindamycin	29	<0.25 to ≥4	2	2	10 (34.5)	18 (62.1)	1 (3.4)
Erythromycin	29	≤0.25 to >16	0.5	1	18 (62.1)	6 (20.7)	2 (6.9)
Cefazolin	29	≤8 to ≥64	≤8		15 (51.7)	6 (20.7)	8 (27.6)
Daptomycin	22	≤0.12 to 4	2	4	8 (36.4)	12 (54.5)^c^	
Cefotaxime	22	4 to ≥64	16	32	2 (9.1)	18 (81.8)	2 (9.1)
Ampicillin/sulbactam	25	≤0.25 to >8	4	>8	1 (4)	–	24 (96)
Ampicillin	29	0.5 to >8	>4	>4	0	–	29 (100)
Ceftazidime	22	32 to >64	>64	>64	0	0	22 (100)

^a^Minimum inhibitory concentration

^b^MIC at which 50 or 90 % of tested isolates are inhibited

^c^This isolates were interpreted as “not susceptible” because the breakpoint was only set for susceptible

### Antibiotic treatment

In the appropriate empirical therapy group, piperacillin-tazobactam (3 cases), vancomycin (2 cases), or ampicillin–sulbactam, cefmetazole, clindamycin, amikacin, or cefoperazone–sulbactam (1 case each) was used. In the inappropriate empirical therapy group, cefepim (6 cases), ampicillin–sulbactam, cefazolin, ceftriaxone/cefotaxim (3 cases each), ceftazidime (2 cases), cefotiam, or clindamycin (1 case each) was used, and in vitro testing showed that the *B. cereus* isolated on blood culture was resistant. One patient was not treated empirically with antibiotics. The duration of antimicrobial therapy did not differ between the two groups (Table 3). All patients, but one, were changed to an appropriate antibiotic agent by 5 days.

**Table 3:** Antibiotic empirical therapy and outcomes of *Bacillus cereus* bloodstream infection

Variable	All patients (n = 29)	Empirical therapy that was deemed		*P* value
		Appropriate (n = 9)	Inappropriate (n = 20)	
Duration of appropriate antibiotic treatment median (days, range)	15 (7–41)	13 (7–20)	16.5 (7–41)	0.050
Early defervescence (patients)	10	6	4	0.032*
Survival at 4 weeks (patients)	27^a^	8/9 (88.9 %)	19/19 (100 %)	0.321

* Statistically significant (p < 0.05)

^a^One patient was excluded in this analysis because of transfer to another hospital

### Clinical outcome

In terms of the primary outcome, no significant difference in all-cause mortality at 4 weeks after onset of bacteremia was seen between the groups (Table 3). However, the secondary outcome of early defervescence was achieved significantly more often in the appropriate empirical therapy group. Central venous catheters were all removed in both groups. The presence of blood cultures that became negative was confirmed in 6 patients of the appropriate therapy group and 17 patients of the inappropriate therapy group. However, the intervals of taking blood cultures differed depending on the cases.

## Discussion

The present data show that the BSI of *B. cereus* was mostly caused by venous catheter-related infections and that appropriate empirical therapy is important to achieve early clinical resolution in *B. cereus* bloodstream infection, although the appropriateness of therapy does not affect mortality.

In this study, cases detected *B. cereus* in more than two sets of blood cultures were enrolled as definite BSI and accounted for 14.3 % of *B. cereus*-detected cases. In our hospital, the rate of obtaining two sets of blood cultures was around 60 % at the beginning of the study period, and it increased to 75 % at the end. Although a further increase of the two-set rate may enable us to obtain more cases and uncover the detailed clinical picture of *B. cereus* BSI, the criterion used to select the definitive *B. cereus* BSI cases was more than two sets of positive blood cultures according to the BSI guideline [23]. *B. cereus* bacteremia was reported to be underestimated as contamination [24, 25], and it has recently been increasingly recognized that some cases of *B. cereus* bacteremia are definite BSIs [15, 17].

Bacteremia due to other bacteria, such as *Staphylococcus aureus* and extended-spectrum beta lactamase-producing Gram-negative bacteria, caused higher 30-day mortality in patients with various comorbidities than *B. cereus* bacteremia even when empirical therapy was inappropriate [26, 27]. In the present study, only one patient died in the appropriate therapy group, and no patient died in the inappropriate therapy group, indicating that *B. cereus* might be less virulent to humans. However, the appropriateness of therapy still has benefit for patients with *B. cereus* BSI, because early defervescence was significantly related to appropriate empirical antibiotic therapy.

Which antibiotic agents are appropriate for bacteremia due to *B.cereus*? In the present study, the *B. cereus* isolated was frequently not susceptible to empirical therapy, but an early treatment response was related to susceptible empirical therapy.

In this study, all isolates were resistant to ampicillin, and almost all were resistant to cephalosporins. All were sensitive to carbapenems. This could be explained by the fact that *B. cereus* is genetically resistant to all beta-lactams except carbapenems [3]. However, some reports showed carbapenem-resistant *B. cereus* bacteremia [28, 29], and some in vitro studies showed that *B. cereus* possesses metallo-beta lactamase genetically [30, 31]. Thus, further study is needed to determine whether carbapenems could be one of the choices for empirical treatment of *B. cereus* BSI.

Fluoroquinolones and clindamycin are also possible if a *B. cereus* isolate is susceptible. The present data showed a resistance rate of 65.5 % (19/29 cases) for clindamycin and 10.3 % (3/29) for levofloxacin. Horii et al. reported that 26 *B. cereus* isolates from blood cultures showed MIC90 of levofloxacin as 4 (CLSI’s breakpoint of levofloxacin was ≤2 as sensitive) [11]. When empirical therapy is started with these agents, the susceptibility pattern should be considered.

All isolates showed good susceptibility to vancomycin according to CLSI’s breakpoint (MIC ≤4 as sensitive). Other reports [11, 22] also showed no vancomycin-resistant *B. cereus* isolated from both clinical samples and environmental samples. Given that a main cause of *B. cereus* BSI is catheter-related infection, vancomycin is suitable for empirical treatment of *B.cereus* BSI, because the common causative pathogens of catheter-related infection are Gram-positive bacteria including *S. aureus* and coagulase-negative *Staphylococci*. However, BSI caused by *B. cereus* with vancomycin-reduced susceptibility (MIC 4 μg/mL) was reported in neonates [32]. We should pay attention to changes in susceptibility to vancomycin.

Other optimal choices for Gram-positive bacteria, daptomycin and linezolid, are options in *B.cereus* BSI. In the present study, the MIC90 of linezolid was 1, and all isolates were considered susceptible. In contrast, more than half of the *B. cereus* showed the MIC of daptomycin >1, although breakpoint of daptomycin was ≤1 as susceptible. The MIC of daptomycin was tested by microdilution assay. Luna et al. [22] reported that the MICs of daptomycin were 0.25–1 using the E test for 42 strains of *B.cereus* collected from the environment. The differences in the MICs might be due to differences in methodology or differences in whether they were clinical isolates or not. An in vitro study reported that a gene that gave daptomycin resistance was activated under swarming condition [33], and that spores were resistant to daptomycin until they germinated [34]. Thus, further study of the measurement of daptomycin MIC and the use of daptomycin for treatment is needed.

This study has some limitations. First, whether antimicrobials were susceptible was determined by antimicrobial breakpoints of *B. cereus* in CLSI’s guideline M45A2E [19]. However, for some antimicrobials for which breakpoints have not been determined, breakpoints of *S. aureus* in M100 [21] were used with reference to a previous report [22]. Although breakpoints of these species were similar for various classes of antimicrobials such as cephalosporins, vancomycin was slightly different. This may be limitation of this study because undefined breakpoints of *B. cereus* may differ from those of *S. aureus* already defined in M100 [21]. We hope that various antimicrobial breakpoints of *B*. *cereus* would be set based on the abundant experience with the treatment of *B.cereus* infection. Second, *B. cereus and B. thuringiensis* were not distinguished in this study, because the automated identification system was used. Although *B. thuringiensis* is basically a harmless pathogen to humans, the extremely rare case with *B. thuringiensis* infection might have been missed. Third, the study was performed retrospectively based on clinical records, and the number of cases was small. To determine the most effective therapy for *B. cereus* BSI, more large-scale and prospective studies should be performed.

## Conclusions

The definite BSI of *B.cereus* accounted for 14.3 % of *B.cereus*-detected cases, and they were mostly caused by venous catheter-related infections. Early clinical resolution was related to appropriate empirical antibiotic therapy. Vancomycin is optimal for empirical therapy of *B. cereus* BSI, but further clinical data are needed.
